# Host and Environmental Factors Affecting the Intestinal Microbiota in Chickens

**DOI:** 10.3389/fmicb.2018.00235

**Published:** 2018-02-16

**Authors:** Jannigje G. Kers, Francisca C. Velkers, Egil A. J. Fischer, Gerben D. A. Hermes, J. A. Stegeman, Hauke Smidt

**Affiliations:** ^1^Department of Farm Animal Health, Faculty of Veterinary Medicine, Utrecht University, Utrecht, Netherlands; ^2^Laboratory of Microbiology, Wageningen University & Research, Wageningen, Netherlands

**Keywords:** gut microbiota, poultry, confounding factors, microbiome, gut health, 16S rRNA

## Abstract

The initial development of intestinal microbiota in poultry plays an important role in production performance, overall health and resistance against microbial infections. Multiplexed sequencing of 16S ribosomal RNA gene amplicons is often used in studies, such as feed intervention or antimicrobial drug trials, to determine corresponding effects on the composition of intestinal microbiota. However, considerable variation of intestinal microbiota composition has been observed both within and across studies. Such variation may in part be attributed to technical factors, such as sampling procedures, sample storage, DNA extraction, the choice of PCR primers and corresponding region to be sequenced, and the sequencing platforms used. Furthermore, part of this variation in microbiota composition may also be explained by different host characteristics and environmental factors. To facilitate the improvement of design, reproducibility and interpretation of poultry microbiota studies, we have reviewed the literature on confounding factors influencing the observed intestinal microbiota in chickens. First, it has been identified that host-related factors, such as age, sex, and breed, have a large effect on intestinal microbiota. The diversity of chicken intestinal microbiota tends to increase most during the first weeks of life, and corresponding colonization patterns seem to differ between layer- and meat-type chickens. Second, it has been found that environmental factors, such as biosecurity level, housing, litter, feed access and climate also have an effect on the composition of the intestinal microbiota. As microbiota studies have to deal with many of these unknown or hidden host and environmental variables, the choice of study designs can have a great impact on study outcomes and interpretation of the data. Providing details on a broad range of host and environmental factors in articles and sequence data repositories is highly recommended. This creates opportunities to combine data from different studies for meta-analysis, which will facilitate scientific breakthroughs toward nutritional and husbandry associated strategies to improve animal health and performance.

## Introduction

In recent years several articles have been published on the intestinal microbiota composition of chickens and its associations with production and health (Nava et al., 2007; Brisbin et al., 2008; Kohl, 2012; Stanley et al., 2012a; Yeoman et al., 2012). For instance, some studies have described differences in bacterial species abundance for broilers with high vs. low growth and feed efficiency (Stanley et al., 2012a; Singh et al., 2014). Another important topic in microbiota research is *Clostridium perfringens*-associated necrotic enteritis that can cause severe production losses and disease in broilers, and can cause foodborne illness in humans (Hook et al., 1996; Van Immerseel et al., 2004). Necrotic enteritis is associated with perturbations in microbiota composition, but whether these are cause or effect of *C. perfringens* proliferation remains unclear (Stanley et al., 2012b; Antonissen et al., 2016; Moore, 2016). Also, the ban on antibiotic growth promoters in the European Union has prompted research into developing alternative nutritional strategies aiming at stimulation of beneficial microbiota in chickens (Stanley et al., 2014). These examples illustrate that it is essential to increase our biological understanding of the host-microbe interactions, which may eventually result in effective strategies to promote sustainable poultry production.

**FIGURE 1:**
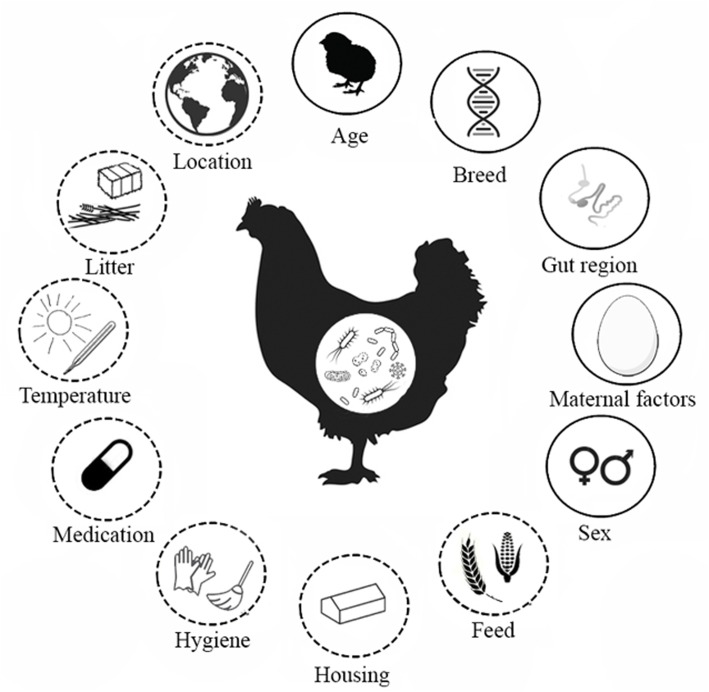
Factors that affect the intestinal microbiota composition of chickens. Factors found in the literature that determine the development of the intestinal microbiota in broiler chickens. Solid line indicates host characteristics, dashed line indicates environmental factors. The gut regions comprise the crop, proventriculus, gizzard, duodenum, jejunum, ileum, caeca, large intestine and cloaca. Maternal factors include horizontal transmission, vertical transmission and maternal antibodies.

Although much progress has been made in this rapidly expanding research field, researchers using next generation sequencing (NGS) tools have reported large differences in microbiota composition across and within studies (Stanley et al., 2013; Brooks et al., 2015; Reardon, 2016). A meta-analysis of gut microbiota studies across different avian species showed that a large factor contributing to the observed variation in avian intestinal microbiota composition was the study itself (Waite and Taylor, 2014). Within the same study, differences in intestinal microbiota composition may also occur across independent poultry trials, even when the research conditions are carefully controlled and intended to be similar across trials (Stanley et al., 2013; Thibodeau et al., 2017). Comparison of the outcomes of microbiota studies might be hampered by differences in technical aspects, biological variation within and between hosts, and environmental factors (Lozupone et al., 2013; Brooks et al., 2015; Laukens et al., 2016). Multiplex sequencing of 16S ribosomal RNA (rRNA) gene amplicons, which is often used to profile the composition of the intestinal microbiota, is associated with technical variation. Differences in the sequencing platforms used, the choice of PCR primers and corresponding region to be sequenced, the number of PCR cycles, DNA extraction protocols, and the storage of samples can create variation in outcomes between studies. These technical factors have been reviewed previously (Pissavin et al., 2012; Lozupone et al., 2013; Brooks et al., 2015; Hermes et al., 2015; Laukens et al., 2016; Allali et al., 2017) and are therefore beyond the scope of this review. The aim of this review is to provide an overview of poultry-specific host and environmental factors that affect the composition of the intestinal microbiota of poultry, and to create awareness of confounding factors in poultry microbiota studies. Knowledge of these factors will enable the improvement of design and reproducibility of outcomes of poultry microbiota studies. An overview of biological and environmental factors potentially influencing chicken microbiota composition described in the literature is shown in **Figure 1**. In the following sections, known effects of host characteristics and environmental factors on intestinal microbiota will be described, followed by a discussion of the potential implications of these confounding factors for microbiota research in poultry.

## Host Characteristics Influencing Intestinal Microbiota in Poultry

### Development of the Chickens

One-day-old broiler chicks already carry a community of microorganisms in their intestinal tract (Ballou et al., 2016). Microorganisms can be acquired in the pre-hatching phase, directly from the mother in the oviduct of the hen (Gantois et al., 2009) or from the environment through the pores in the eggshell (Cason et al., 1994; Roto et al., 2016). In a recent publication it was shown that broiler eggs contaminated with cecal microbiota on the egg surface of other birds reduced the bird-to-bird variation in the cecal microbiota composition after hatch but not the composition itself (Donaldson et al., 2017). This means that the cecal microbiota on the egg surface resulted in more similarity between the microbiota samples of the individual broilers, but the microbiota of the donors associated with high or low performance was not actually transferred to the newly hatched broilers. After hatch, the young chicks might be colonized before arriving at the farm by microbiota from the environment at the hatchery or during transport (Shapiro et al., 1949; Pedroso et al., 2005).

The microbiota composition may also be influenced by maternal antibodies supplied through the yolk. Maternal antibodies can provide protection against colonization by certain pathogens generally until 2 weeks post-hatch (Grindstaff et al., 2003; Hamal et al., 2006), and this may affect the chicks’ intestinal microbiota. In mammals it is known that maternal antibodies can affect the interaction between intestinal bacteria and the immune system (Cebra, 1999). Although the mechanism behind the interaction of bacteria and the immune system is not exactly clear, the altered development of the immune system in germ-free animals suggests that it is at least partly shaped by the microbiota (Williams, 2014).

**FIGURE 2:**
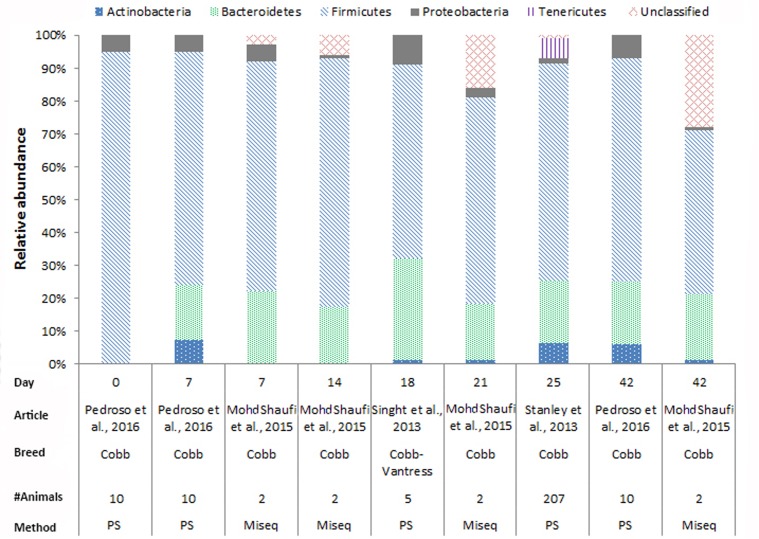
The composition of the cecal microbiota in Cobb broilers at phylum level. General composition of the cecal microbiota in Cobb broilers across different ages, from control groups, not exposed to specific treatments. The data is from four different studies, based on 16S rRNA 454 pyrosequencing (PS), *n* = 3 and MiSeq sequencing *n* = 1. Pedroso et al. (2016), Figure 7, stacked bar chart A was used. Mohd Shaufi et al. (2015), Figure 4, the last four bars were used. Singh et al. (2013), the data from Figure 1 was used. Stanley et al. (2013), the data from MG-RAST was used to combine the data from Figure 6.

**FIGURE 3:**
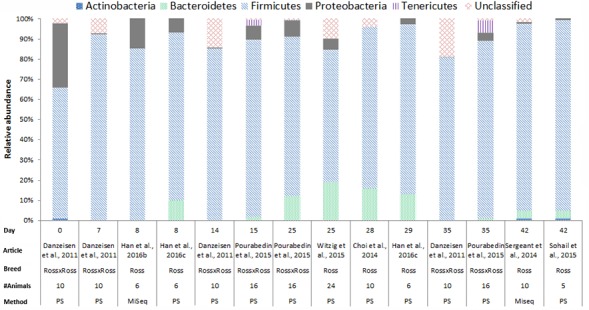
The composition of the cecal microbiota in Ross broilers at phylum level. General composition of the cecal microbiota in Ross broilers across different ages, from control groups, not exposed to specific treatments. The data is from eight different studies, based on 16S rRNA 454 pyrosequencing (PS), *n* = 6 and MiSeq sequencing *n* = 2. Danzeisen et al. (2011), the data from the supporting information Supplementary Table S1 was used. Han et al. (2016b), the data from Figure 6, the first bar of the histogram was used. Han et al. (2016c), the data from Figure 7, pie chart A and C was used. Pourabedin et al. (2015), the data from the supplementary data, Supplementary Figure S1A was used. Witzig et al. (2015), the data from the Supplementary Data, Supplementary Table S4 was used. Choi et al. (2014), data from Figure 1 was used. Sergeant et al. (2014), the data from the Supplementary Data 1 was used. Sohail et al. (2015), the data from Supplementary Table 2 was used.

**FIGURE 4:**
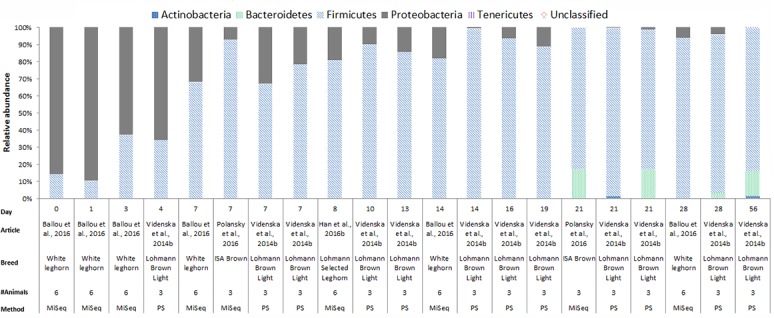
The composition of the cecal microbiota in layer-type chickens at phylum level. General composition of the cecal microbiota in laying hens across different ages, from control groups, not exposed to specific treatments. The data is from four different studies, based on 16S rRNA 454 pyrosequencing (PS), *n* = 1 and MiSeq sequencing *n* = 4. Ballou et al. (2016), data from Figure 2B was used. Videnska et al. (2014b), data from the Supplementary Data S1 for ages 7, 14, 21, 28, and 56, data from the Supplementary Data S2 for ages 4, 7, 13, 16, and 19, and data from the Supplementary S3 data for age 21 was used. Polansky et al. (2016), data from Figure 1, bar 1 and 3 from the histogram was used. Han et al. (2016b) the data from Figure 6, the second bar of the histogram was used.

In chickens the intestinal microbiota richness, i.e., the number of different microbial taxa, increases during the first weeks of life (Gerard et al., 2008; Danzeisen et al., 2011; Ballou et al., 2016), while the individual variation in microbiota composition decreases as the chickens age (Crhanova et al., 2011). A compilation of 16S rRNA gene amplicon sequencing data from cecal samples of two different broiler breeds (meat production) and layer-type chickens (egg production) shows variation at the phylum level across studies, and at different time points (**Figures 2**–**4**). This compilation is based on a systematic literature search. However, the limited number of articles with 16S rRNA gene amplicon sequencing data, and the large methodological differences between poultry studies did not allow for accurate re-analyses of original raw data to provide figures that would represent a true meta-analysis of studies. Therefore only the relative abundance at phylum level of the chickens in groups not exposed to specific treatments (control groups) is summarized to illustrate some general differences in microbiota development with regard to breed and age. *Firmicutes* were the most abundant phylum across the two broiler breeds throughout the production period from 0 to 42 days of age (**Figures 2**, **3**). It is striking that *Firmicutes* were found to be the most abundant phylum on day 0 in meat-type chickens whereas *Proteobacteria* were most abundant in layer-type chickens (**Figure 4**). On day 0 the relative abundance of *Proteobacteria* in layer-type chickens was above 85% (**Figure 4**) (Ballou et al., 2016), whereas in meat-type chickens this phylum only accounted for approximately 30% (Danzeisen et al., 2011) (**Figure 2**), and 5% (Pedroso et al., 2016) (**Figure 3**). *Firmicutes* become the most abundant phylum also in layer-type chickens from day 7 onward (**Figure 4**). For humans it has been shown that facultative anaerobic *Proteobacteria* are the most abundant phylum in the first period of life (Reinhardt et al., 2009; De Filippo et al., 2010; Schwartz et al., 2012), which is also seen in laying hens (Videnska et al., 2014b; Ballou et al., 2016) but not in broilers. The variation in colonization pattern between layer-type and meat-type chickens might be explained by the differences in exposure to microbiota, husbandry factors, and feed composition, but biological differences between these chicken types are most likely to play an important role as well and will be discussed in the next section.

### Chicken Type and Breed

The genetic background of the host has been recognized as a factor that might influence intestinal microbiota composition (Benson et al., 2010; Org et al., 2015; Schokker et al., 2015; Han et al., 2016b). Considerable physiological differences exist between layer-type and meat-type chickens. Over decades, breeding programs have selected laying hens for maximal egg production and broilers for maximal meat production. This has resulted in large differences in growth, with an average body weight of laying hens of 450 g compared to 2800 g at 6 weeks of age in broilers^1^. These chicken breeding programs seem to have affected intestinal physiology (Uni et al., 1996) and immune function (Simon et al., 2014). Morphological differences in the intestinal tissue between laying hens and broiler chickens with respect to villus height, villus width, and crypt depth influence the intestinal absorptive area and have been associated with the higher body weight of broilers (Uni et al., 1996). Moreover, it has been shown that the expression of IgA, IgM, and IgY in the ileum is higher in broilers compared to laying hens (Simon et al., 2014). These and other differences in intestinal physiology and immune system development between laying hens and broiler chickens are likely to influence microbiota composition and vice versa. Studies on differences between broilers and laying hens with regard to microbiota composition are scarce. To our knowledge, only two studies compared the microbiota composition between broilers and laying hens. It should be noted, however, that the first study was done with 3-week-old broilers and 62-week-old laying hens (Videnska et al., 2014a). This large age difference, as well as the difference in exposure to microbes in the housing environment and substantial differences in the composition of the diet for broilers and laying hens, may also have influenced microbiota composition, which hampers conclusions on the effect of chicken type (Videnska et al., 2014a). In the second study, as expected, differences in the development of local immunity and the colonization pattern of commensal bacteria between chicken types were found, and these differences were also shown to significantly alter the response to inoculation with *Campylobacter* (Han et al., 2016b). This indicates that differences in chicken breeds or genetic lines can impact important study outcomes.

In addition to biological differences between layer-type and meat-type chickens, there are also differences within chicken breeds of the same chicken type. As a previous observational study has shown, broiler breed was a factor associated with colonization by antibiotic resistant strains of *Escherichia coli* (Persoons et al., 2011), and in an experimental study it was shown that different broiler breeds significantly differed in disease susceptibility to necrotic enteritis (Jang et al., 2013). In a study with different broiler breeds, hatched in the same hatchery, it was shown that each breed also had its own unique ileum microbiota composition at the age of 20 days (Kim et al., 2015). In 20-day-old Cobb broilers, *Bacteroidetes* were found in the ileum content, but were absent in Ross broilers. In turn, in Ross broilers, *Actinobacteria* were found in the ileum content, but not in Cobb broilers (Kim et al., 2015). Similar results were found in other studies, i.e., absence of *Bacteroidetes* and presence of *Actinobacteria* in Ross broilers at 21 days of age (Nakphaichit et al., 2011) and 25 days of age (Pourabedin et al., 2015), and Cobb broilers without *Actinobacteria* but with *Bacteroidetes* at 23 days of age (Mohd Shaufi et al., 2015). In contrast, in another recent study a relative abundance of 22% of *Bacteroidetes* was reported in the ileum of 18-day-old Ross broilers (Han et al., 2016a). The presence of *Bacteroidetes* in the latter study and absence in the other studies may be caused by inevitable differences in diet or other experimental conditions, the younger age at sampling, differences in sequencing technology; as pyrosequencing vs. Illumina MiSeq, or the differences in the primers used.

We compiled the data of studies for which 16S rRNA gene amplicon sequencing data of cecal samples was available for two broiler breeds (**Figures 2**, **3**). This compilation shows that in cecal samples *Actinobacteria* are present in all four Cobb studies (100%) and in three out of eight Ross studies (38%), and that *Bacteroidetes* are present in all four Cobb studies (100%) and in six out of eight Ross studies (75%) (**Figures 2**, **3**). These figures might suggest that breed influences the microbiota composition, but it is more likely that Cobb and Ross broilers had a different exposure to microbiota due to differences in parent flock or due to differences in the immune responses caused by differences in the genetic background (Emam et al., 2014; Schokker et al., 2015). Furthermore, it should be noted that, unfortunately, many articles on chicken microbiota data do not contain information about the breed (Qu et al., 2008; Corrigan et al., 2015; Lim et al., 2015; Schokker et al., 2015; Li et al., 2016; Oakley and Kogut, 2016; Lin et al., 2017).

Within certain broiler breeds, there is also a distinction between low and high body weight lines. As several studies have revealed, broilers from lines with low and high feed conversion ratio (FCR) show differences in their bacterial communities. In fecal samples, broiler lines with low FCR, indicating a more efficient use of feed for growth, showed higher counts for *Lactobacillus* compared to broilers lines with high FCR (Zhao et al., 2013; Meng et al., 2014; Mignon-Grasteau et al., 2015). Broiler line comparison in another study showed that the composition of the microbiota differed while microbial diversity did not, which might suggest that different chicken lines harbor different microorganisms for the same intestinal function (Schokker et al., 2015). The mechanisms behind the variation in intestinal microbiota between different broiler lines remain unclear, but it has been suggested that genetic background and the immune system influence establishment of gut microbiota after hatch (Schokker et al., 2015). Commercial selection programs for high production may result in co-microevolution of the microbiota and immune system of the host (Yang et al., 2017), although other factors, such as differences in exposure to microbial communities, cannot be excluded.

### Sex

In poultry, sex difference is part of the disparate production system, because layer-type chicken flocks predominantly consist of hens, whereas in broiler flocks males and females are often raised together. Broiler males generally have a higher growth rate and lower FCR than broiler females. Differences in bacterial communities between male and female broilers are also influenced by non-growth related factors, because no differences in growth rate were observed until day 21, whereas already at day 3 differences were observed in the intestinal microbiota composition (Lumpkins et al., 2008). In this study, the intestinal microbiota communities, determined by denaturing gradient gel electrophoresis (DGGE) of PCR-amplified 16S rRNA gene fragments, showed less than 30% similarity between male and females (Lumpkins et al., 2008). Another study, where female and male broilers (age 22 and 42 days) were compared using quantitative PCR (qPCR), showed differences in abundance of *Lactobacillus salivarius*, *L. crispatus*, *L. aviarius*, and *E. coli* in their ceca (Torok et al., 2013). These are four out of the five potential performance-related bacteria of the qPCR format used (Torok et al., 2013). In a study on intestinal microbiota composition in chickens of 245 days of age and different broiler lines, i.e., a high (HW) and low body weight (LW) line, the relative abundance of 48 microbial species was significantly different between sexes (Zhao et al., 2013). Furthermore, there was a significant interaction between genotype and sex. In HW lines, males and females had 30 species of bacteria that were different between them, and LW lines 17 species (Zhao et al., 2013).

In animal studies, often only males are used to create a stable baseline model that is not affected by cyclical reproductive hormone levels (Zucker and Beery, 2010). An interaction between probiotic treatment and sex for *Bifidobacterium* was found in 42-day-old broilers (Mountzouris et al., 2015). These results reinforce that the sex of a chicken might be a confounding factor. Many broiler and microbiota studies contain only data from males (Dumonceaux et al., 2006; Burkholder et al., 2008; Guardia et al., 2011; Hasan and Adem, 2011; La-Ongkhum et al., 2011; Stanley et al., 2012a; Akbarian et al., 2014; Goodarzi Boroojeni et al., 2014; Huff et al., 2015; Ruiz et al., 2015) or the sex of the broilers is unknown (Stanley et al., 2012b; Corrigan et al., 2015; Oakley and Kogut, 2016). This sex bias in literature might influence our understanding of the microbiota development in chickens and therefore the sex of the chicken should always be reported.

### Sampling the Gastrointestinal Tract of Chickens

The gastrointestinal tract (GIT) regions consists of the crop, proventriculus, gizzard, duodenum, jejunum, ileum, caeca, large intestine and cloaca. The GIT regions have different functions that impact microbiota dynamics and should be considered when determining the sampling protocol and study design. Differences in composition and abundances of bacteria between the different GIT regions have been reviewed in detail previously (Yeoman et al., 2012; Stanley et al., 2014; Deusch et al., 2015). The different sections of the GIT have their own specific function in the digestion of feed, suggesting that there are differences in requirements for the types of microbiota that need to be present in each part. The crop primarily stores and pre-processes feed for further digestion (Richardson, 1970). For example, crop samples have been observed to show large differences in microbiota composition between individual broilers on the same diet (Sekelja et al., 2012; Choi et al., 2014). To illustrate, in one study with three individual 28-day-old broilers, the relative abundances of *Firmicutes* amounted to 95, 40, and 32%, of *Proteobacteria* 5, 55, and 19%, of *Bacteroidetes* 0, 3, and 36%, and of *Actinobacteria* 0, 2, and 13% for the three broiler chickens (Choi et al., 2014). The large individual variation in this study may have been related to the time between feeding and sampling. This variable will be discussed in more detail in the section about feed access. The gizzard mechanically grinds feed and acts as a microbial barrier due to its low pH (Stanley et al., 2014), the duodenum receives digestive enzymes from the bile- and pancreatic ducts, and the main function of the ileum is the absorption of nutrients. Those three regions, however, are all dominated by *Lactobacillus* species (Konsak et al., 2013; Stanley et al., 2014).

The main function of the cecum is fermentation of nutrients (Clench and Mathias, 1995). From microbiota data of individual broilers it is known that there is more variability between individual ileum and cloaca samples than between ceca samples (Pissavin et al., 2012; Choi et al., 2014). The cecum is the part of the GIT with the highest microbial richness and is mainly colonized by anaerobic microorganisms (Salanitro et al., 1974; Videnska et al., 2013). The cecal microbiota is more diverse, has a greater richness, and is more stable compared with microbiota residing in the ileum (Gong et al., 2007; Owens et al., 2008; Stanley et al., 2014). In addition, an adequate sample size for a study depends on the type of samples as well. The high individual variation in crop samples compared to cecal samples will result in a lower number of cecal samples needed to find a potential difference.

Intestinal samples of chickens can only be acquired post-mortem, and therefore, in many studies a less invasive method of sampling is preferred. Fecal samples and collection of cecal droppings have been used to determine intestinal microbiota composition. Cecal droppings reflect broilers’ cecal microbiota, whereas fecal droppings do not (Pauwels et al., 2015; Stanley et al., 2015). Cecal droppings are difficult to collect because they are usually more easily trampled by the chickens and are produced less frequently than fecal droppings, with one cecal dropping for every seven to eight fecal droppings (Williams, 1995). Consequently, for comparisons between groups or studies, the location from which the intestinal samples originate should be taken into account to avoid misinterpretation of results.

## Environmental Factors Influencing Intestinal Microbiota in Poultry

### Biosecurity Level

In poultry production, it has been suggested that compared to the situation where a chicken is hatched by the mother hen, the relatively high hygiene levels of hatcheries have an effect on the development of the GIT and immune system. It is suggested that this is due to a delayed exposure to a ‘healthy’ microbiota (Bailey, 2010), which is comparable to the ‘hygiene hypothesis’ postulated for humans (Lashner and Loftus, 2006). Moreover, the high hygiene levels within hatcheries may also result in variable intestinal microbiota between batches of newly hatched chickens. It has been hypothesized that their intestinal bacterial community is shaped rather randomly and is quite heterogeneous due to exposure to bacteria from a variety of environmental sources after hatch, rather than colonization by maternally derived bacteria (Stanley et al., 2013). These environmental sources include people handling the chicks, transport crates, the first feed and the litter in the poultry house. In broilers raised in isolators, it was shown that the intestinal morphology was altered with shorter villi, more shallow crypts and a reduced production of acidic mucin compared with conventionally raised broilers (Forder et al., 2007), which might result in a different microbiota composition. In studies with other animals, for example in pig studies it has been shown that the intestinal development in high hygiene environments, such as isolators, negatively impacts a normal succession of the intestinal microbiota because it influences the expression of large numbers of immune-related genes (Mulder et al., 2011), and reduces the microbiota diversity compared to piglet siblings raised on a farm (Inman et al., 2010).

### Housing

Studies in humans have reported that individuals who live together show less variation of the intestinal microbiota compared to a group of random individuals (Yatsunenko et al., 2012; Schloss et al., 2014). In animal studies, a living-together effect, also referred to as a cage effect, is well-known, especially for animals that are coprophagic such as mice (McCafferty et al., 2013; Laukens et al., 2016). Since chickens are coprophagic as well, a cage effect is likely to occur in chicken studies. To avoid cage effects and to prevent uncontrolled intake of particles and feathers containing potentially influencing intestinal microbiota (Meyer et al., 2012), some researchers house the birds individually (Zhao et al., 2013; Org et al., 2015). Cage was also a factor in a study on *C. perfringens*, which showed that the variation in *C. perfringens* count tended to be smaller between birds from the same pen (Hofshagen and Kaldhusdal, 1992). Furthermore, as researchers recently proposed, different experimental units may differentially shape especially the non-dominant microbiota in broilers (Ludvigsen et al., 2016).

Also, the type of production system can influence microbiota composition. In a study comparing organic farms to conventional farms, a higher number of *C. perfringens* was found in ileum and caecum samples of broilers from organic farms (Bjerrum et al., 2006). In this case, the researchers suggested that this difference might be due to the antimicrobial drug salinomycin, applied as coccidiostat in the conventional feed, which has antibiotic properties that can affect the intestinal microbial composition (Bjerrum et al., 2006). Moreover, they found lower counts of *Enterobacteriaceae* and higher lactobacilli numbers in the ileal content of the birds raised on the organic farms (Bjerrum et al., 2006). Access to an outdoor range was demonstrated to enrich *Bifidobacterium* in ceca and ileum in Ross broilers (Gong et al., 2008), and resulted in a higher proportion of *Bacteroidetes* in the cecum and a lower *Firmicutes*/*Bacteroidetes* ratio in Dagu chickens (Xu et al., 2016). In the ceca of Dagu chickens housed in free-range systems, a higher abundance of bacteria associated with functions involved in amino acids and glycan metabolic pathways was observed (Xu et al., 2016). In the previous example, access to range may have altered the composition of the microbiota, but the timing of access to the range may be important as well. When access to range occurred during the last 4 weeks only, instead of from the beginning of the production period onward, no change in the richness of the broiler intestinal microbiota was found (Gong et al., 2008). Furthermore, the broiler density in a flock was also shown to affect the performance and the intestinal bacterial community (Beloor et al., 2010; Guardia et al., 2011). In a flock with a stocking density of 17 birds per m^2^ a decrease in growth performance and bacterial composition in the cecal samples was found, compared to a stocking density of 12 birds per m^2^ (Guardia et al., 2011). This effect was more pronounced in the first half of the broiler production period. However, whether this was a direct effect of the alterations in microbiota or due to other health and management problems associated with increased stocking densities remained unclear.

### Litter

In poultry farming, litter is a mix of fecal and composted bedding material. Litter is an important environmental factor since chickens peck and forage in the litter. Litter is also used to collect samples to determine the intestinal composition of a flock. It has been demonstrated that the microbiota composition of litter samples collected from different production systems clustered with the corresponding microbiota composition of cecal samples (Mancabelli et al., 2016), suggesting that microbiota is exchanged between the chickens and the litter.

Depending on the litter type, litter quality and litter management the bacterial composition of chickens varies (Torok et al., 2009; Pan and Yu, 2014). It has been shown that litter type can affect the intestinal microbiota composition, for example birds raised on softwood sawdust vs. chopped straw showed significant differences in cecal microbial communities at 28 days of age (Torok et al., 2009). Also, it has been shown that female broilers grow slower on paper litter than on wood litter (Torok et al., 2011). This stresses the importance of the choice of litter material for microbiota studies, as it might affect interventions.

The quality of litter has, in several studies, been associated with the performance of the chickens (Welfare Quality, 2009; de Jong et al., 2014). Litter quality has been observed to vary also within the same poultry house, with for example, higher moisture content of litter underneath nipple drinkers (Dumas et al., 2011). Wet litter was found to have greater microbiota (alfa) diversity than dry litter, and this might influence the intestinal microbiota as well (Dumas et al., 2011; Oakley et al., 2013). Although in general litter samples of the same flock do not share many taxa with fecal samples, wet litter was more similar to fecal samples than dry litter (Oakley et al., 2013).

Reused litter may harbor pathogens from the previous flock (Stanley et al., 2004). In broilers reared on 7-day-old fresh litter the ileal microbiota was dominated by *Lactobacillus* spp., whereas in broilers reared on reused litter a group of unclassified *Clostridiales* were the dominating bacteria in the ileal microbiota (Cressman et al., 2010). In the litter the microbial (alpha) diversity between fresh litter and reused litter became similar at day 42 (Cressman et al., 2010). Another study showed that as litter aged, litter microbial diversity decreased (Pedroso et al., 2013), whereas the opposite tendency was observed for chicken intestinal microbiota.

### Feed Access

After the first ingestion of feed after hatch a large increase in bacterial numbers in the chicken intestine can be observed (Shapiro and Sarles, 1949). Access to feed stimulates villus heightening and increased generation of cells in the crypt in young chicks (Gonzalez-Moran et al., 1985). In young chicks, delay in access to feed affects the development of the intestinal surface area (Uni et al., 1998; Lamot et al., 2014), and therefore potentially also the microbiota composition (Flint et al., 2012). Feed withdrawal later in life has also been associated with changes in microbiota composition (Burkholder et al., 2008; Vossen et al., 2009). Temporary feed withdrawal can result in an increased intestinal pathogen colonization (Thompson et al., 2008), for instance with *Salmonella* (Burkholder et al., 2008). After 6 h of feed deprivation, large changes in the bacterial community were observed in the proximal part of the GIT (Vossen et al., 2009). Daily cycles of light and darkness, feeding rhythm, or temperature affect eating behavior of animal hosts which creates a daily rhythm of the digestive system. As a consequently, many bacteria experience substantial environmental changes during the day, due to eating behavior of animal hosts, which is referred to as a bacterial circadian clock (Johnson et al., 2017). In a mouse study, cyclical changes in the intestinal microbiota from feeding/fasting rhythms added to the intra-individual variation (alfa diversity) of intestinal microbiota (Zarrinpar et al., 2014). Therefore, it is important that the time of feeding and/or feed deprivation and the moment of sampling are kept similar between birds or groups and are documented in scientific articles. Unfortunately, details on the duration of fasting before sampling are often not described.

### Climate and Geographical Location

The local climate in a poultry house is an important factor that is well-known to influence the performance of chickens. The number of studies describing the effects of climate on microbiota, using 16S rRNA gene amplicon sequencing, are, however, limited. For heat stress, however, some studies are available that describe both the large effects on performance and alterations of the microbiota composition of broilers (Lan et al., 2004; Sohail et al., 2015). These alterations can lead to susceptibility to *E. coli* (Laudadio et al., 2012) and can contribute to increased intestinal colonization by *Salmonella* (Burkholder et al., 2008; Soliman et al., 2009). When birds experienced stress due to exposure to higher temperatures for 24 h, greater changes were shown to occur in the ileal content compared to cecal samples, indicating that the microbiota in the ileum may be more sensitive to changes than the cecal microbiota (Burkholder et al., 2008).

The geographic location may affect the climate in the poultry house and as a consequence may influence the intestinal microbiota of chickens (Videnska et al., 2014a; Zhou et al., 2016). Although temperature in poultry houses is often controlled, broiler production may decrease because of the unfavorable influence of a hot environment (Laudadio et al., 2012). Especially when high ambient temperatures are combined with high relative humidity, chickens can experience heat stress. This may be the reason why in one flock in Austria in the years 2003–2006 and 2013 no seasonal effect was identified (Sofka et al., 2015). Recently, a between-sample (beta) diversity analysis did not show specific clustering based on the different geographical locations. However, effects of the geographical location were detectable when comparing species richness and intra-individual diversity (Siegerstetter et al., 2017). For many studies, geographical location and its effects on the climate the birds are exposed to are unknown. It is therefore often difficult to evaluate to what extent these factors may influence the research results.

## Implications of Confounding Factors Affecting the Intestinal Microbiota in Chickens

The aim of this review was to provide an overview of host and environmental factors that affect the composition of the intestinal microbiota of poultry, to create awareness of confounding factors in poultry microbiota studies. We summarized the currently available knowledge regarding potential confounding factors separately, but of course, many of those factors cannot be seen independently. This review emphasizes the relevance of comprehensive documentation and reporting, as well as control of relevant host and environmental factors and molecular approaches in poultry microbiota studies, as previously suggested for studies with humans and mammals (Kilkenny et al., 2010; Laukens et al., 2016).

Of the factors that influence poultry microbiota composition (**Figure 1**), antibiotics and feed composition are well-known for their effects on performance and the intestinal microbiota. Antibiotics and feed composition are often the main interventions that are the focus of a given study and were not discussed in this review, as our aim was to show which host and environmental factors that are *not* under investigation act as confounders, and may unintentionally have a large impact on the study outcome. For example, rather than the antibiotic treatment, a stronger effect on the composition of the microbiota was attributed to the environment in which chickens were raised, i.e., battery cages vs. floor pens (Pedroso et al., 2006). Furthermore, there are examples of studies that indicated an unexpected lack of differences in intestinal microbiota composition between diet interventions (Van Der Hoeven-Hangoor et al., 2013; Park et al., 2015), but did show clustering for different GIT regions, age groups and cages (Park et al., 2015; Ludvigsen et al., 2016). Consequently, not taking confounding factors properly into account with study design and data analysis might explain why antibiotics or a feed intervention does not show effects or an unrepeatable effect on the intestinal microbiota composition. Thus, host characteristics and environmental factors can have a large impact on conclusions that can be drawn from experiments and field studies.

Using knowledge of relevant confounding factors to improve study designs is an essential prerequisite to being able to generate data that will facilitate thorough understanding of the phylogeny and composition of the microbiota, and functionality of host-microbiota interactions. Although controlling for confounders is not always possible, detailed recording and reporting of these factors should be considered as an integral and essential part of each study. Providing details on study variables such as feed composition, feed access, feed changes, feed deprivation, medications (preventive and therapeutic), vaccines, age, sex, hygiene protocols, housing systems, litter type, flock density, housing temperature and location in the methodology part of publications, will improve the repeatability, the reproducibility, and the interpretation of chicken microbiota studies.

**FIGURE 5:**
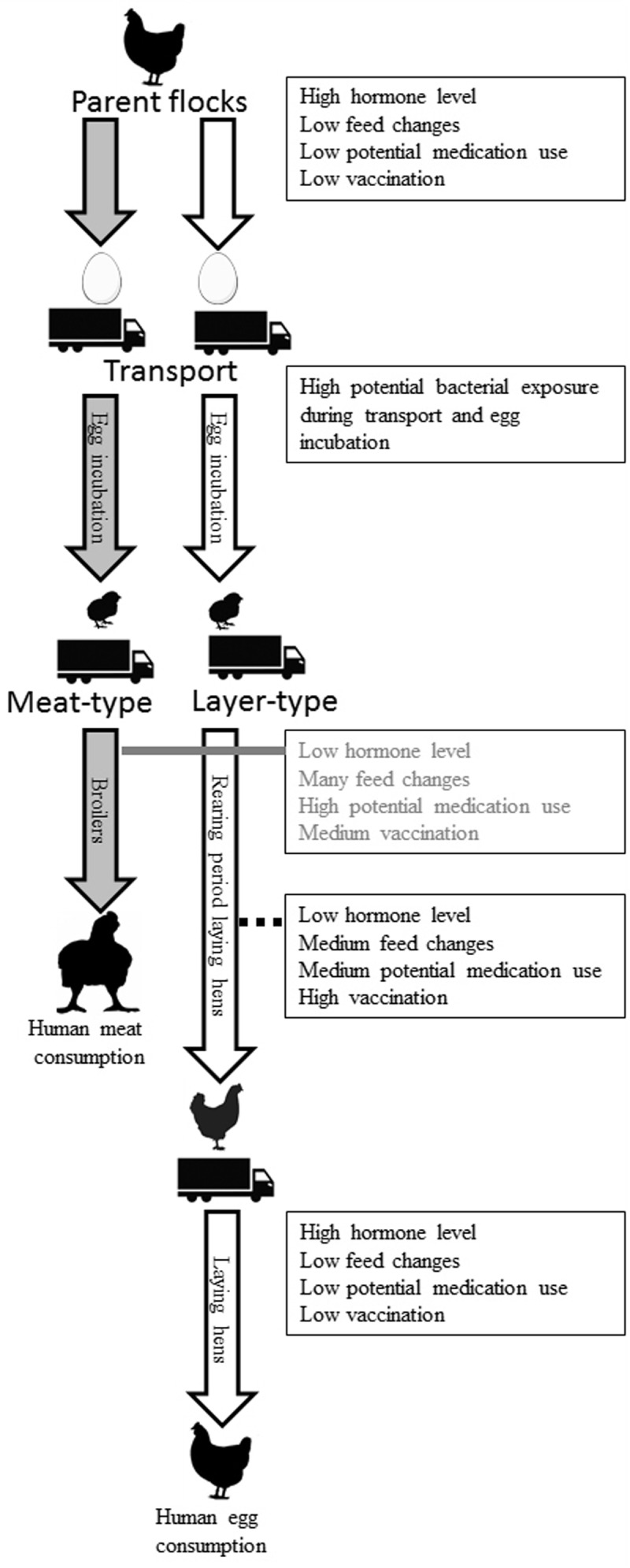
Known and potential factors that affect the intestinal microbiota composition of chickens during life. These factors can have short- and long-term effects and may even originate from the hatching stage or a previous generation. Grandparent flocks and the rearing period of the parent flock are not included in the figure. Layer- (white arrow) and broiler-type (gray arrow) have different production systems and are therefore displayed separately.

During the life of a chicken, many of the host and environmental factors discussed in this review can exert their respective effects on chickens. **Figure 5** provides a compilation of known and potential effects on the intestinal microbiota composition of chickens, illustrating that many factors can have both short- and long-term effects and may even originate from the hatching stage or a previous generation. Layer-type and meat-type chickens have different production systems and are therefore displayed separately. The influences of hormonal changes due to the reproduction cycle are limited in broilers, due to their short lifespan, and also limited in the first part of the rearing period of layers. Near the end of the rearing period and the first part of the laying period, development of the reproductive organs and the start of egg production may affect GIT microbiota composition, as shown in mice (Org et al., 2016). Exposure to bacteria early in life, before, during and shortly after hatching, during transport or at the start of the rearing period (for layers) or production period (for broilers) has a potentially large impact on microbiota composition and immune development for both the short- and the long-term (Maynard et al., 2012). No or a delayed feed access, for example during transport to a farm, also can influence intestinal microbiota composition (Simon, 2016). Nevertheless, if this delayed feed access effect is biologically relevant in terms of stimulation and functionality of the immune system is still unclear. It is clear that perturbation of early life microbial colonization has long-term effects on immune development (Simon et al., 2016; Schokker et al., 2017).

The rearing period is associated with more feed changes, more preventive and therapeutic treatments and vaccinations than the laying period. In broilers, many feed changes, treatments, and vaccinations occur in a very short lifespan of approximately 6 weeks. It is known that different *Salmonella* vaccines used in broilers can change cecal microbiota composition (Park et al., 2017). This most likely also happens in the rearing period of laying hens. Since it is currently unknown how all those potential factors influence intestinal microbiota composition of chickens, further investigation is needed.

In **Figures 2**–**4**, 16S rRNA gene amplicon sequencing data was combined from different studies, although it should be noted that there is still limited 16S rRNA gene amplicon sequencing data available for one-day-old to 7-day-old broilers. In addition, the sample size of most studies is also limited, especially in the laying hen studies where the sample size is 3–6 birds. Another important observation that follows from these data is that in some studies, 30% (**Figure 2**) to 20% (**Figure 3**) of 16S rRNA multiplex amplification data has remained unclassified. To increase our biological understanding of host-microbe interactions these unknown microbes need to be identified. Consequently, there is still limited evidence that the colonization pattern of layer- and meat-types is different. Despite the limited availability of data and methodological differences between studies, it seems safe to conclude that layer- and meat-type chickens follow a different colonization pattern compared to mammals. For example, in human babies, a period has been identified were members of the phylum *Actinobacteria* are present in a high proportion (Reinhardt et al., 2009; De Filippo et al., 2010; Ottman et al., 2012; Schwartz et al., 2012). This period with a high proportion of *Actinobacteria* is neither observed in laying hens (Videnska et al., 2014b; Polansky et al., 2016; Ballou et al., 2016), nor in broilers (**Figures 2**, **3**). In contrast with mammals that drink milk during the first weeks of life, chickens ingest solid feed from the day of hatch onward, which might explain the variation in colonization pattern between mammals and birds. Another possible explanation for the observed large differences between data of mammalian studies and poultry studies, is that in mammalian studies often fecal samples are used, whereas in chicken studies cecal samples are most often collected.

## Concluding Remarks

Comprehensive analyses of intestinal microbiota will lead to better understanding of dynamics in microbial community structure and function, which will increase our understanding of intestinal health in poultry. Hence, research aimed at identifying biologically relevant characteristics of a healthy poultry microbiota, for instance as a foundation for nutritional and husbandry associated strategies to replace antimicrobial drugs, is both promising and challenging. It has been shown that microbiota studies have to deal with many hidden host and environmental variables, which are not all known. Therefore it is essential to be aware of the large impact the choice of study designs has on the results and thus on the interpretation of the outcomes of studies into the intestinal microbiota. Furthermore, providing details on study variables and sequence data repositories creates opportunities to combine data from different studies for meta-analysis, which will facilitate scientific breakthroughs toward innovative microbiota-inspired intervention strategies.

## Author Contributions

JK, FV, EF, JS, and HS initiated the project. JK searched the databases for potentially eligible articles based on their titles and abstracts and wrote the paper. FV, EF, GH, JS, and HS contributed to the development of the manuscript as a whole by giving constructive feedback on the manuscript during its preparation. All authors gave approval of the manuscript for publication.

## Conflict of Interest Statement

The authors declare that the research was conducted in the absence of any commercial or financial relationships that could be construed as a potential conflict of interest.

## Footnotes

1 http://en.aviagen.com/assets/Tech_Center/Ross_Broiler/Ross-Broiler-Handbook-2014i-EN.pdf, http://www.hyline.com/UserDocs/Pages/36_COM_ENG.pdf, http://www.lohmanngb.co.uk/files/Classic-Colony-Manual-28-Mar-2011.pdf
